# First Detection of Deformed Wing Virus (DWV) and Acute Bee Paralysis Virus (ABPV) in Central Hungary in European Hornet (*Vespa crabro* Linnaeus, 1758)

**DOI:** 10.3390/ani15243565

**Published:** 2025-12-11

**Authors:** János Gál, Árisz Ziszisz, Márton Hoitsy, Míra Mándoki, Krisztina Bali, Lilla Dénes, Enikő Fehér, Ákos Jerzsele, Gábor Halász, Eszter Kaszab

**Affiliations:** 1 Department of Exotic Animal-, Wildlife-, Fish- and Honeybee Medicine, University of Veterinary Medicine, H-1078 Budapest, Hungary; gal.janos@univet.hu (J.G.);; 2 Budapest Zoo and Botanical Garden, H-1146 Budapest, Hungary; 3 Department of Pathology, University of Veterinary Medicine, H-1078 Budapest, Hungary; 4 Department of Microbiology and Infectious Diseases, University of Veterinary Medicine, H-1078 Budapest, Hungary; 5 National Laboratory for Infectious Animal Diseases, Antimicrobial Resistance, Veterinary Public Health and Food Chain Safety, H-1078 Budapest, Hungary; 6 National Laboratory of Virology, Szentágothai Research Centre, University of Pécs, H-7624 Pécs, Hungary; 7 Department of Pharmacology and Toxicology, University of Veterinary Medicine, H-1078 Budapest, Hungary; 8 Department of Bioinformatics, One Health Institute, University of Debrecen, H-4032 Debrecen, Hungary

**Keywords:** acute bee paralysis virus (ABPV), deformed wing virus (DWV), honeybee viruses, *Vespa crabro*, Hungary

## Abstract

This study aimed to investigate the presence of known bee viruses in the European hornet (*Vespa crabro*, Linnaeus, 1758), a species recognized as a bee predator in Hungary. Several viruses affecting honeybees (*Apis mellifera*, Linnaeus, 1758), such as deformed wing virus (DWV), sacbrood virus (SBV), chronic bee paralysis virus (CBPV), and acute bee paralysis virus (ABPV), have been documented in various wasp species. For instance, DWV has been frequently isolated in *Vespa orientalis* (Linnaeus, 1761), and ABPV has been detected in *V. orientalis*. Additionally, viruses like Kashmir bee virus (KBV) and Black queen cell virus (BQCV) have been confirmed in other wasp species such as *Vespula germanica* and *Vespa velutina*. Despite this, data on virus presence in *V. crabro* remain limited. Between August and October 2023, we tested 40 adult *V. crabro* workers, collected from Kiskunlacháza and Vácduka, for viral infections using polymerase chain reaction (PCR). Our results confirmed the presence of genetic material from DWV and ABPV infection in adult workers of the European hornet, which showed no morphological alterations. This study provides the first detection of DWV (in Hungary) and ABPV in *V. crabro*, contributing to our understanding of virus transmission pathways in wasp species and their potential impact on bee populations.

## Simple Summary

This study investigated the presence of honeybee-associated viruses in the European hornet, a known bee predator. While certain viruses have been detected in other wasp species, limited data existed regarding their occurrence in European hornets. To address this gap, researchers collected 40 adult hornets from Hungary between August and October 2023 and tested them for viral infections. Molecular analysis identified genetic material from two viruses known to affect honeybees, one associated with wing deformities and another linked to paralysis. Notably, the infected hornets exhibited no visible signs of disease. This study represents the first confirmed detection of these viruses in European hornets in Hungary, contributing to a broader understanding of virus transmission across insect species. These findings highlight the ecological role of *V. crabro* in the spread of pollinator pathogens, threatening honeybee populations and ecosystem stability, and underscore the need for targeted monitoring and mitigation efforts.

## 1. Introduction

The European hornet (*Vespa crabro*, Linnaeus, 1758), one of the largest representatives of the folding-winged wasps (Vespidae) in Hungary, is a social, colony-forming wasp species widely distributed across Europe, with dense populations in certain regions [1]. These wasps initiate nest-building in the spring, with colony expansion continuing throughout the summer [2]. In autumn, only the fertilized queens hibernate over the winter period. In spring, they start building the nest. The nest, consisting of hexagonal cells, is constructed to house the larval stages of the first generation, which are nurtured for approximately 21–24 days before maturing into adult workers. After that, the pupated and mature workers assume the role of caring for the larvae, allowing the colony to grow further [2]. Essentially, they feed their larvae with other insect species, including honeybees (*Apis mellifera*). Towards the end of summer, they prefer the carcasses of dead animals and ripe, sweet fruits such as grapes [1].

Globally, several viral infections have been identified in honeybees, including acute bee paralysis virus (ABPV), Israeli acute paralysis virus (IAPV), Kashmir bee virus (KV), chronic bee paralysis virus (CBPV), deformed wing virus (DWV), sacbrood virus (SBV), and black queen cell virus (BQCV) [3]. These viruses, known to affect bees, have also been detected in other arthropods. For example, DWV can be detected not only in honeybees but also in many other arthropod species, including solitary (one species) and eusocial wasp species (seven species), ants (two species), butterflies (three species), and bumblebees (eleven species) [4]. Other studies have also reported the viral infection in several wasp species, which are also known as bee predators [5], with specific attention drawn to invasive wasps as vectors of these bee viruses [6]. Notably, DWV was one of the most frequently detected viruses in *Vespa orientalis* [7]. An examination was also carried out on the larvae of this wasp species, in which DWV was also the most frequently detected virus [8]. In one report, the clinical appearance of DWV, such as shortened and deformed wings, was also observed in *Vespa crabro,* and the complete genome sequence of DWV was determined [9,10].

CBPV, which occurs in apiaries worldwide, was also found in two ant species (*Camponotus vagus*, Scopoli, 1763; *Formica rufa*, Linnaeus, 1761) in addition to bees [11]. In bees, besides paralysis symptoms, this virus can also cause the loss of the hair covering of the abdomen [3]. The virus could not be detected in adult and larval wasps examined [7,8].

The presence of ABPV [3], which often causes symptomless infection in bees but can also result in wing asymmetry and paralysis, was detected with a frequency of 17.2% in larvae and 63.3% in adults of the species *Vespa orientalis* [7,8].

IBPV, known to cause the loss of the hair covering the abdomen and paralysis in bees, has also been found in *V. velutina*, a natural bee predator, with evidence suggesting the virus may replicate in this wasp species [12].

SBV, which induces molting disorders in honeybee brood, has been detected in *V. orientalis* with a prevalence of 34.4% in larvae and 3.3% in adults [3,7,8]. Interestingly, SBV, previously known in bees, was also detected in the species, the Giant resin bee (*Megachile sculpturalis*, Smith, 1853), in an Italian study [13]. BQCV, which causes the disease and death of bee broods, is widespread worldwide and was detected in 43.3% of adult *V. orientalis*, while in the larva stage of this wasp species, it was detected in 24.1% [3,7,8].

Kashmir bee virus (KBV), which is associated with *Varroa destructor* mites in domestic honeybees and is often the cause of hive depopulation, may also be present in other species [3]. Based on different studies, the virus has been identified in other species, such as the Asian hornet (*Vespa velutina*) in Italy and the German wasp (*Vespula germanica*) [14,15]. KBV is listed as a potential wasp pathogen in a New Zealand study [16]. The pathogen was detected in 3.3% of *V. orientalis* adults, which could not be confirmed in camouflage [7,8].

To date, references to the European hornet (*Vespa crabro*) being infected with pathogenic bee viruses are notably scarce in international literature. Currently, only the presence of DWV has been identified in the case of *Vespra crabro* in Italy. Despite growing concern over viral transmission among pollinators, evidence of pathogenic bee viruses in the European hornet remains limited. This knowledge gap limits our understanding of the species’ role in pathogen ecology.

## 2. Materials and Methods

A total of 40 European hornet (*Vespa crabro*) samples were collected in 2023 in Hungary (Kiskunlacháza and Vácduka) (Table 1). Solitaire samples were captured during their resting period on fruits at night, as the collection of live hornets during active daylight hours presents substantial safety risks. There were no known beehives or apiaries in the immediate vicinity of the sampling locations.

The European hornet samples in 500 µL phosphate-buffered saline (PBS) were homogenized using a mortar and pestle. To enrich viral nucleic acids from the homogenized European hornets, samples were centrifuged at 3000 rpm for 5 min. Viral nucleic acid was extracted from the supernatant using MagCore^®^ Plus II instrument and MagCore^®^ Viral Nucleic Acid Extraction Kit (202) (RBC Bioscience, New Taipei City, Taiwan) according to the manufacturer’s instructions.

For the detection of different viruses, QIAGEN OneStep RT-PCR Kit (Qiagen, Hilden, Germany) was used with specific primer sets, listed in Table 2. The reaction was performed according to the manufacturer’s instructions in a final volume of 25 µL. The reaction mixture comprised 1 µL enzyme mix, 4 U RiboLock RNase Inhibitor (Thermo Scientific™, Waltham, MA, USA), the primer pair at 0.6 µM concentration, and 2 µL template RNA. The PCR cycling conditions were 50 °C for 30 min and 95 °C for 15 min, followed by 35 cycles of 94 °C for 30 s, 57 °C for 30 s, and 72 °C for 1 min; the final extension lasted for 5 min at 72 °C. Positive controls for each virus were derived from our previous study [17].

The PCR product was run on a 1.5% agarose gel (Top Vision Agarose, Thermo Scientific™, Waltham, MA, USA) and stained with Invitrogen SYBR™ Safe DNA Gel Stain (Thermo Scientific™, Waltham, MA, USA). The PCR product was excised and extracted from the gel using the QIAGEN QIAquick Gel Extraction Kit (Qiagen, Hilden, Germany). To confirm the results, Sanger sequencing was performed for selected samples by a third-party service provider (Eurofins Biomi Ltd., Gödöllő, Hungary).

For phylogenetic analysis, we built datasets for Deformed wing virus and Acute bee paralysis virus based on the partial sequence of RdRp. Sequence alignments were created with the MAFFT plugin of Geneious Prime^®^ v.2024.0.5. Maximum-likelihood trees were inferred with MEGA-X, using the TN92+G model for each dataset and applying 100 bootstrap replicates [18]. Sequence identity values were calculated using Geneious Prime software v.2024.0.5.

## 3. Results and Discussion

Table 3 contains the results of the samples collected in our research between August and October 2023.

During the external morphological examination of the hornets captured for this study, no abnormal deviations were observed on the head, thorax, or abdomen, nor on the legs and wings, all of which were intact. The morphological examination ruled out macroscopic signs of DWV. The clinical examination assessed movement coordination and grooming behavior to evaluate potential effects of ABPV.

**Figure 1:**
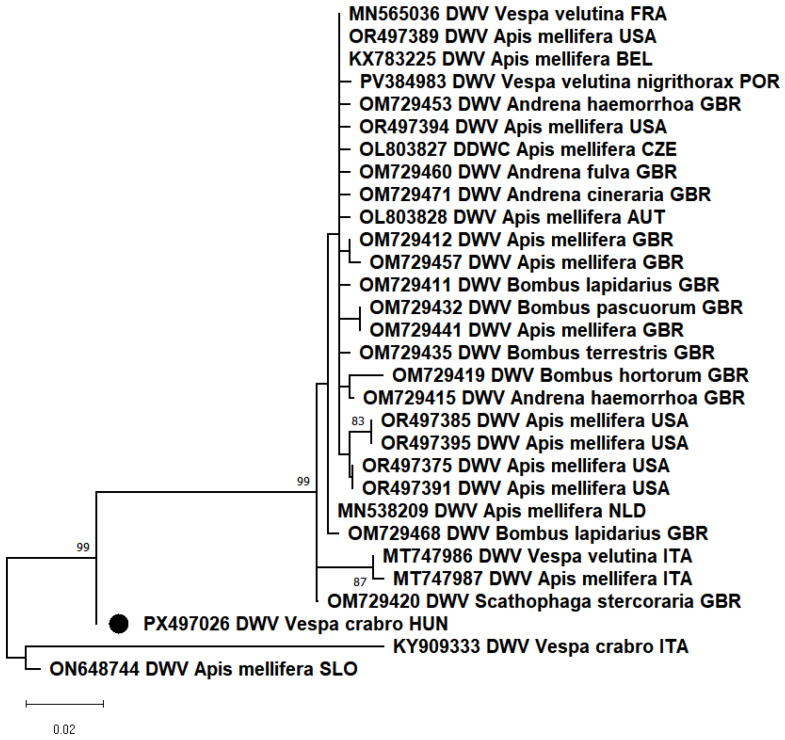
Based on the partial nucleotide sequences of the RNA-dependent RNA polymerase gene of deformed wing virus (DWV), a maximum-likelihood phylogenetic tree was created with MEGA-X software, T92+G model, 100 bootstrap repetitions. The strain we have described is indicated by a black circle.

PCR results showed that most of the samples from Kiskunlacháza and Vácduka were positive for the deformed wing virus (DWV). This is the first confirmed detection of DWV in apparently healthy *V. crabro* workers in Hungary. Previously, such cases had only been reported in Italy [9,10]. In other studies, DWV is described as one of the most frequently isolated viruses in both adults and larvae of *Vespa orientalis* [7,8]. The partial RdRp segment of our sample showed 97.21% nt identity with the closest reference sequence (ON648744), which was detected in *Apis mellifera* from Slovenia. Our sample showed only 90.52% nt identity with the other DWV detected in *Vespa crabro*. In the phylogenetic analysis, the DWV_HUN-1 (PX497026) strain clustered in a separate branch (Figure 1).

**Figure 2:**
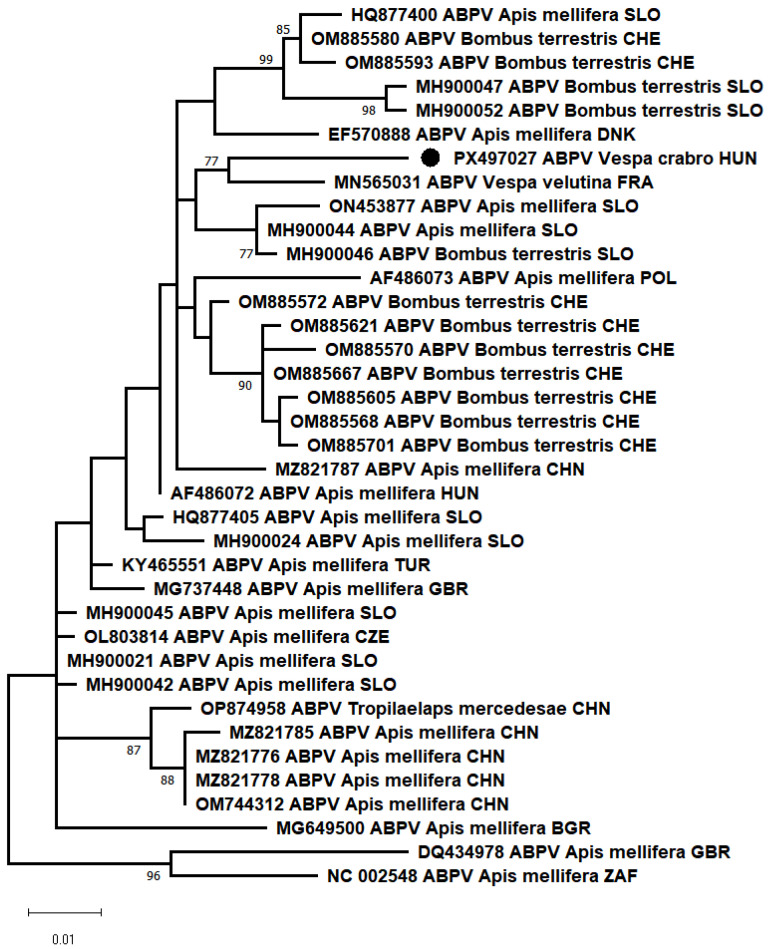
Maximum-likelihood phylogenetic tree based on the partial nucleotide sequences of the RNA-dependent RNA polymerase gene of acute bee paralysis virus (ABPV), made with the MEGA-X software, T92+G model, 100 bootstrap repetitions. The strain we have described is indicated by a black circle.

Additionally, PCR results showed that most of the samples from both collection sites were positive for the acute bee paralysis virus (ABPV), marking the first detection of ABPV in healthy *V. crabro* workers. In other research, the genetic material of ABPV was detected with a frequency of 17.2% in larvae and 63.3% in adults of the species *Vespa orientalis* [7,8]. The partial RdRp of our sample showed 97.08% nt identity with the closest reference sequence (MN565031), which was detected from the Asian hornet (*Vespa velutina nigrithorax*, Buysson, 1905). In the phylogenetic analysis, the ABPV_HUN-1 (PX497027) strains clustered together with the closest reference sequences (Figure 2).

DWV and ABPV infection were simultaneously detected in both collection locations (Kiskunlacháza and Vácduka) in the examined hornets. While the Kashmir bee virus (KBV) has previously been confirmed in *Vespa velutina*, *Vespula germanica*, and *V. orientalis*, we did not detect KBV in any of the *V. crabro* specimens [7,8,14,15,16]. We could not confirm the IBPV infection during the examination of our *V. crabro* specimens, although the presence of the virus has been reported in *V. velutina*, where it may even replicate [12]. BQCV has already been detected in *V. orientalis* imagos (43.3%) and larvae (24.1%), but we could not identify it in *V. crabro* workers. Furthermore, CBPV has already been detected in imagoes and larvae of two ant species (*Camponotus vagus* and *Formica rufa*) and *V. orientalis*; in contrast, all samples in our tests were negative [7,8,11]. Although SBV has been described in *V. orientalis* larvae and imagos, as well as in the species *Megachile sculpturalis*, we could not detect this virus in *V. crabro* workers [7,8,13].

The surveillance for certain viral infections, such as deformed wing virus (DWV), black queen cell virus (BQCV), acute bee paralysis virus (ABPV), sacbrood virus (SBV), chronic bee paralysis virus (CBPV), and Israeli acute paralysis virus (IAPV), was carried out in Hungarian apiaries in several years. Most recently, in the spring of 2024, screening tests were performed in 26 domestic and 3 foreign apiaries and on free-collected bee samples to identify the aforementioned virus strains [17]. However, bee predator species have not yet been examined to determine whether these species are asymptomatic carriers of the viruses. We are currently conducting a comprehensive series of diagnostic PCR tests to clarify how the European hornet (*V. crabro*), the most important predator of bees in Hungary, becomes infected with the most important viruses known to affect bees.

## 4. Conclusions

This study reports the first detection of acute bee paralysis virus (ABPV) in the European hornet (*Vespa crabro*) and the first record of Deformed wing virus (DWV) in this species in Hungary. Both viruses were found in adult hornets that showed no symptoms at two different locations. This suggests that *V. crabro* may be a silent carrier (transmitter) of honeybee-related viruses. Phylogenetic analyses showed close relationships between the viral sequences from the hornets and those found in honeybees and other wasp species. This points to possible pathways for transmission between species.

These findings increase our understanding of the range of hosts or transmitters, as well as the geographic spread of bee viruses. They highlight the ecological role of *V. crabro* in the potential spread of pollinator pathogens. Further research should examine whether they are acquired through eating contaminated prey or environmental exposure, and whether these viruses can reproduce in hornet hosts. Such studies will be important for understanding how viruses spread among pollinators and predators and what this means for honeybee health and the stability of ecosystems.

## Figures and Tables

**Table 1:** Collected samples.

Number	Place of Collection	Date of Collection	Type of Collection	Species	Copy
1.	Kiskunlacháza	August 2023	free collection	European hornet (*Vespa crabro*)	10
2.	Vácduka	September 2023	free collection	European hornet (*Vespa crabro*)	25
3.	Kiskunlacháza	October 2023	free collection	European hornet (*Vespa crabro*)	5

**Table 2:** Primers of honeybee viruses were used for the test.

ID	Primer Sequences	Pathogens	Fragment Size
A	5′ TTTGCAAGATGCTGTATGTGG 3′	DWV (Deformed wing virus)	395 bp
A2	5′ GTC GTGCAGCTCGATAGGAT 3′		
B	5’ GGATGAAAGGAAATTACCAG 3’	SBV (Sacbrood virus)	426 bp
B2	5’ CCACTAGGTGATCCACACT 3’		
C	5’ AGTTGTCATGGTTAACAGGATACGAG 3’	CBPV (Chronic bee paralysis virus)	455 bp
C2	5’ TCTAATCTTAGCACGAAAGCCGAG 3’		
D	5’ TGAGAACACCTGTAATGTGG 3’	ABPV (Acute bee paralysis virus)	452 bp
D2	5’ ACCAGAGGGTTGACTGTGTG 3’		
E	5’ GGACGAAAGGAAGCCTAAAC 3’	BQCV (Black queen cell virus)	424 bp
E2	5’ ACTAGGAAGAGACTTGCACC 3’		
F	5’ GATGAACGTCGACCTATTGA 3’	KBV (Kashmir bee virus)	414 bp
F2	5′ TGTGGGTTGGCTATGAGTCA 3′		
G	5′ AGATTTGTCTGTCTCCCAGTGCACAT 3′	IAPV (Israeli acute paralysis virus)	475 bp
G2	5′ AGACACCAATCACGGACCTAC 3′		

**Table 3:** Results of PCR tests from the pooled samples in different months.

Date of Collection	DWV	SBV	CBPV	ABPV	BQCV	KBV	IAPV
	Deformed Wing Virus	Sacbrood Virus	Chronic Bee Paralysis Virus	Acute Bee Paralysis Virus	Black Queen Cell Virus	Kashmir Bee Virus	Israeli Acute Paralysis Virus
August 2023, *n* = 10	+	-	-	+	-	-	-
September 2023, *n* = 20	+	-	-	+	-	-	-
September 2023, *n* = 5	-	-	-	-	-	-	-
October 2023, *n* = 5	+	-	-	+	-	-	-

## Data Availability

The partial RdRp sequences of ABPV and DWV were deposited in GenBank with the following accession numbers: PX497026 and PX497027.

## Abbreviations

The following abbreviations are used in this manuscript:

ABPV	Acute bee paralysis virus
BQCV	Black queen cell virus
CBPV	Chronic bee paralysis virus
DWV	Deformed wing virus
IAPV	Israeli acute paralysis virus
KV	Kashmir virus
SBV	Sacbrood virus
